# First Molecular Detection of SARS-CoV-2 in Sewage and Wastewater in Ghana

**DOI:** 10.1155/2024/9975781

**Published:** 2024-03-25

**Authors:** Ewurabena Oduma Duker, Evangeline Obodai, Seth Offei Addo, Lorreta Kwasah, Edna Serwah Mensah, Emmanuel Gberbi, Abraham Anane, Keren O. Attiku, Jessica Boakye, Gayheart Deladem Agbotse, Angelina Evelyn Dickson, Joseph Ahia Quarcoo, Patience Akosua Darko, Yaw Awuku Larbi, Nana Afia Asante Ntim, Bartholomew Dzudzor, John Kofi Odoom

**Affiliations:** ^1^Virology Department, Noguchi Memorial Institute for Medical Research, University of Ghana, Legon, Accra, Ghana; ^2^Parasitology Department, Noguchi Memorial Institute for Medical Research, University of Ghana, Legon, Accra, Ghana; ^3^Department of Medical Biochemistry, University of Ghana Medical School, University of Ghana, Legon, Accra, Ghana

## Abstract

Severe acute respiratory syndrome coronavirus 2 (SARS-CoV-2) is shed in the stool of infected individuals and can be detected in sewage and wastewater contaminated with infected stool. This study is aimed at detecting the virus and its potential survival in sewage and wastewater in Ghana. The cross-sectional study included samples from 16 validated environmental surveillance sites in 7 regions of Ghana. A total of 354 samples composed of wastewater (280) and sewage (74) were collected from November 2020 to November 2022. Overall, 17% of the samples were positive for SARS-CoV-2 by real-time PCR, with 6% in sewage and 11% in wastewater. The highest number of positive samples was collected from the Greater Accra Region (7.3%) with the least recorded in the Bono East Region (0.6%). Further characterization of the positive samples using the next-generation sequencing (NGS) approach yielded two variants: Alpha (B.1.1.7) and Delta (AY.36). Attempts to isolate SARS-CoV-2 in the Vero cell line were not successful probably due to the low viral load concentrations (Ct values > 35) or prolonged exposure to high temperatures rendering the virus noninfectious. Our findings suggest that SARS-CoV-2 RNA in sewage and wastewater may not be infectious, but the prevalence shows that the virus persists in the communities within Ghana.

## 1. Introduction

A new severe acute respiratory syndrome coronavirus 2 (SARS-CoV-2) was reported to cause the outbreak of COVID-19 in Wuhan, China in December 2019. This SARS-CoV-2 was declared a pandemic by the WHO on March 11, 2020, after the disease was reported in 114 countries. The virus is transmitted chiefly via respiratory droplets through coughing, sneezing, or exhalation as well as fomites [1]. Other alternative transmission routes such as the gastrointestinal tract have also been implicated [2]. Stools from infected patients have been reported to contain viral RNA and as such can be used to determine the presence of the virus in a population [3, 4]. Some reports have also shown that 50% of those who test positive for the virus experience intestinal infections and shed the viral RNA in their faeces [5]. A disturbing aspect is that between 40% and 80% of those who are infected show no symptoms [6, 7]. Mass screening has been recommended, but it is costly and requires the use of experts; hence, alternative cost-efficient methods for early detection/diagnosis of the virus will be essential to curbing infection acquisition and transmission.

SARS-CoV-2 could persist in wastewater for over two weeks under good conditions [8]. The virus and its genetic fragments are present in sewage systems, and this could be an indicator of community-level infections, suggesting that sewage and wastewater could be potential sources of infection. Thus, population-based monitoring of the virus through this medium could play the role of an early warning signal for the (re)emergence of COVID-19 [9]. It is however unclear if sewage and wastewater might contain the infectious virus that could facilitate its potential spread.

As in the polio eradication program where environmental surveillance has been effective in creating supplementary data to complement human surveillance [10, 11], successful detection of SARS-CoV-2 in the environment will be vital in understanding transmission patterns [12, 13]. It will also help to observe changes and trends in circulation at the population level as well as provide information to support public health interventions [14]. On March 12, 2020, the first two cases of COVID-19 were confirmed in Ghana. While Ghana's response to the pandemic has been worth noting, the nation faced challenges as a result of limited resources for testing which adversely influenced SARS-CoV-2 control and elimination in the country.

In Ghana, there is limited information on the environmental spread of SARS-CoV-2. Using environmental surveillance tools to assess community infections of SARS-CoV-2 will initiate a sustainable nationwide monitoring system to complement surveillance in humans. As such, this study sought to investigate sewage and wastewater samples for SARS-CoV-2 viruses and determine their viability and transmissibility.

## 2. Methods

### 2.1. Study Area

This study was cross-sectional, involving sample collection from validated environmental surveillance (ES) sites for poliovirus detection. Samples were collected from 16 sites in Ghana, namely, Akosombo and Koforidua in the Eastern; Agbogbloshie, Shiabu, Nima Freetown, and Legon Campus in the Greater Accra Region; Koblimahgu and Nyanshegu in the Northern Region; Aflao, Hohoe, Kpando, and Togbe Afede Road in the Volta Region; Ahinsine and Asokore Mampong Zongo in the Ashanti Region; Sunyani Zongo in the Bono Region; and Techiman Sisaline in the Bono East Region.

### 2.2. Sample Collection and Processing

Sample collection was from November 2020 to November 2022. Raw sewage and wastewater samples were collected using the grab method of sampling as described by the WHO field guidance for environmental surveillance [15] and shipped on ice to the Regional Reference Polio Laboratory at the Noguchi Memorial Institute for Medical Research. The samples were stored at 4°C and processed within 24 hrs upon receipt in the laboratory.

Sewage and wastewater samples were processed using the two-phase separation method according to the WHO guidelines [15]: briefly, samples were allowed to stand on the bench for about 5 minutes to enable sedimentation of the large solid material. Each raw sample (500 ml) was poured into a tube and centrifuged at 1500 g for 20 min at 4°C. The supernatant was then transferred into a one-liter Erlenmeyer flask. A volume of 287 ml of 29% PEG6000, 39.5 ml of 22% dextran, and 35 ml 5 N NaCl was added to each supernatant obtained and thoroughly mixed by stirring for one hour at 4°C to create a homogenous mixture. The mixtures were poured into separation funnels and staged overnight at 4°C. The lower phase concentrates were harvested into sterile 50 ml centrifuge tubes.

### 2.3. Detection of SARS-CoV-2 RNA by Real-Time PCR and Virus Isolation

Ribonucleic acid (RNA) was extracted from the collected concentrates using a QIAamp RNA kit (QIAGEN, Valencia, CA, USA) according to the manufacturer's instructions. The RNA extracts were used as templates in the RT-PCR assay for SARS-CoV-2 detection using the VERI-Q nCoV-OM detection kit. Each PCR reaction mix consisted of 10 *μ*l of 2x One-Step RT-PCR Master mix, 1 *μ*l Primer/Probe mixture, 1 *μ*l internal positive control, and 8 *μ*l of the RNA template in a total volume of 20 *μ*l. RNA-positive and RNA-negative (nuclease-free water) controls were included in each PCR run. The PCR was performed in an ABI 7500 RT-PCR system (Life Technologies, Grand Island, NY, USA). The cycling conditions were set at 50°C for 10 minutes in 1 cycle, 95°C for 3 minutes in 1 cycle, 95°C for 9 seconds, and 58°C for 30 seconds in 45 cycles.

#### 2.3.1. Virus Isolation

Vero E6 cells (African green monkey kidney, American Type Culture Collection) were cultured using a Dulbecco's minimum essential medium (DMEM) that was supplemented with 10% heat-inactivated FBS, 200 U/ml penicillin-streptomycin, and 200 U/ml L-glutamine and incubated at 37°C/5% CO_2_. The concentrates were filtered using 0.22 *μ*m filters to get rid of debris or sewage remnants that could contaminate the cell lines. Fifty microliters (50 *μ*l) of the filtrate was inoculated unto cultured cells in the microtiter plate alongside a virus control and a cell control and then incubated at 37°C/5% CO_2_. The cells were examined for cytopathic effects (CPE) every 24 hours for 5 days. A second blind passage was done using 50 *μ*l of the previously cultured supernatant and observed every 24 hours for 5 days.

### 2.4. Genetic Characterization of SARS-CoV-2

The PCR-positive samples were subsequently sequenced using Oxford Nanopore's MinION Sequencing at the Noguchi Memorial Institute for Medical Research.

cDNA preparation was done using 2 *μ*l of LunaScript RT SuperMix (New England Biolabs (NEB), Ipswich, MA, United States) and 8 *μ*l of template RNA according to the manufacturer's instruction. Targeted amplification of the SARS-CoV-2 genome was then performed in a Veriti thermal cycler (Applied Biosystems, Bedford, MA, United States) using a master mix of 5x Q5 Reaction Buffer, 10 mM dNTPs, Q5 Hot Start DNA Polymerase, V3 Pool 1/V3 Pool 2, nuclease-free water, and 2.5 *μ*l cDNA template. The cycling conditions were 98°C for 30 s, followed by 35 cycles of amplification at 98°C for 15 s and 65°C for 5 min.

Qubit dsDNA broad range kit from Invitrogen (Waltham, Massachusetts) was used to quantify the DNA amplicons. Subsequently, the Ultra II End-Repair/dA-Tailing Module (New England Biolabs (NEB), Ipswich, MA, United States) was used for the end preparation reaction, while the Next Quick Ligation Module (New England Biolabs (NEB), Ipswich, MA, United States) was used for barcode ligation.

MinION library preparation was performed according to the manufacturer's instructions in the Ligation Sequencing Kit (SQK-LSK109; ONT, Didcot, United Kingdom).

Different barcoded samples were pooled with equal masses. The concentration of each library was measured using a Qubit 4.0 Fluorometer (Invitrogen, Carlsbad, CA, United States), and the volume of each library was then calculated to make an equimolar pool of libraries. The pool of libraries was finally sequenced using the ONT MinION platform (MinKNOW 19.12.5).

The MinION analyzer generated raw data files, and these were imported into the Artic filed bioinformatics pipeline. The sequences were uploaded onto the Pangolin COVID-19 lineage assigner software which assigned the SARS-CoV-2 genome detected to the most likely lineage. Phylogenetic analysis was conducted in MEGA11 using the maximum likelihood method.

### 2.5. Statistical Analysis

All data cleaning and statistical analyses were conducted in STATA version 16.1 (StataCorp, Texas, USA), and graphs were generated using Microsoft Excel. Categorical variables were summarised as frequencies and percentages. A graph was plotted to show the distribution of PCR-positive results from the selected districts over time. The SARS-CoV-2 positivity by source, districts, and months was compared using Pearson's chi-squared test. *p* values of < 0.05 were considered statistically significant.

## 3. Results

### 3.1. SARS-CoV-2 Detection from Sewage and Wastewater

From the seven regions and 16 sites, 354 samples were collected of which 280 (79%) were wastewater and 74 (21%) were sewage (Table 1). The majority of the samples were collected from the Greater Accra Region (*n* = 102, 28.81%) with the least collected from the Bono Region (*n* = 24, 6.78%).

Of the samples collected, 59 (17%) tested positive for SARS-CoV-2 by PCR. The SARS-CoV-2 positivity rate was relatively higher in samples collected from sewage sources (sewage = 27% (20/74) vs. wastewater = 14% (39/280), *p* = 0.013) (Table 1). Furthermore, SARS-CoV-2 positivity from samples collected varied significantly by region (*p* = 0.03) with more positives recorded in Greater Accra (25%), closely followed by the Eastern Region (23%). At least one SARS-CoV-2-positive sample was recorded at each site. Overall, the highest number of SARS-CoV-2 positives was recorded in the Greater Accra Region (*n* = 26) with most observed in Shiabu (*n* = 9) (Table 1). The Eastern Region recorded the second-highest number (*n* = 11) of SARS-CoV-2 positives.

All PCR-positive samples were negative by virus isolation and showed no CPE on culture.

**Figure 1:**
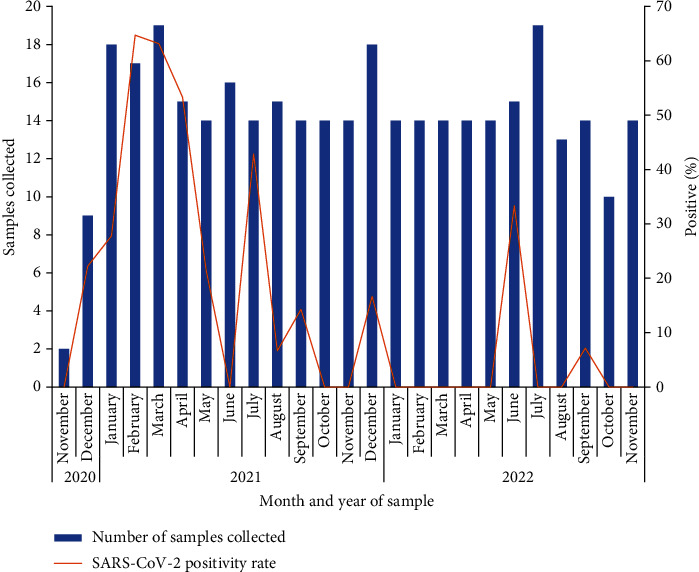
SARS-CoV-2 in sewage and wastewater in Ghana, 2020-2022. Solid blue bars show the number of sewage and wastewater samples (*n* = 354); the red line shows the percentage of samples positive for SARS-CoV-2.

The highest SARS-CoV-2 positivity rates were recorded among samples collected in 2021, around February (Figure 1). It was observed that the number of SARS-CoV-2-positive samples increased from November 2020, peaked in February 2021, and declined until June 2021. An increase was seen again in July 2021 followed by a drop in positivity.

Attempts made to culture the 59 positive samples obtained in this study on Vero cell lines to ascertain the viability/infectivity of the virus proved futile. No cytopathic effect was observed for all 59 positive samples.

### 3.2. SARS-CoV-2 Variants Identified in Sewage and Wastewater Samples, 2020-2022

**Figure 2:**
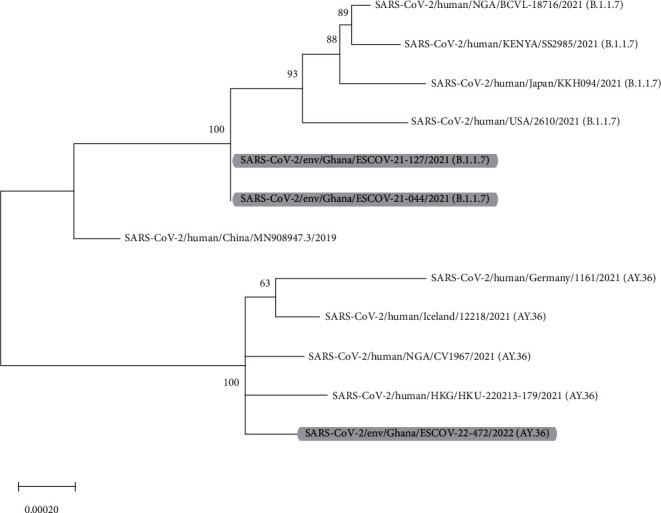
Phylogenetic analysis of SARS-CoV-2 variants from sewage and wastewater in Ghana, 2020-2022. The sequences identified in this study are highlighted.

The first two sequences (B.1.1.7) are clustered with sequences predominantly from American, Asian, and African origin with high bootstrap value (100). These variants were detected in 2021 from the Ashanti Region (Asokore Mampong Zongo) and the Greater Accra Region (Shiabu), respectively. The third sequence (AY.36) was clustered with sequences predominantly from Africa, Europe, and Asia and was detected in 2022 from the Eastern Region (Akosombo site) (Figure 2).

The sequences obtained in this study have been deposited in GISAID: B.1.1.7 (acc. nrs.EPI_ISL_18042705 (ESCOV-21-127/2021) and EPI_ISL_18042704 (ESCOV-21-044/2021)) and AY.36 (acc. EPI_ISL_18042703 (ESCOV-22-472/2022).

## 4. Discussion

This environmental study successfully identified SARS-CoV-2 RNA from sewage and wastewater in Ghana; however, the virus did not grow in culture. This is the first study in Ghana to identify SARS CoV-2, in wastewater and sewage from all study sites across the 7 selected regions in Ghana. An overall 17% SARS-CoV-2 positivity was recorded in our study, which is lower than a study in Italy that found 50% of the wastewater samples to be positive for SARS-CoV-2 [12]. The findings in our study can also be compared to a study in the Czech Republic and Spain that reported SARS-CoV-2 to occur in 11.6% and 12.28% of wastewater samples, respectively [16, 17]. The different positivity rates could be due to the varying sample sizes as the studies in Italy, the Czech Republic, and Spain had sample sizes of less than 100. The difference could also be due to the sample concentration methods used in the studies (direct flocculation vs. two-phase (separation)). The regional variations in the confirmed cases of SARS-CoV-2 in Spain (218,652) [18], Italy (181,228) [12], and the Czech Republic (10,064) [17] compared to Ghana (153,514) [19] could have also influenced the positivity rates. We also observed that SARS-CoV-2 RNA was abundant in sewage (*n* = 74) as compared to wastewater (*n* = 280). This could be attributed to the fact that the sewer systems receive faecal waste from other areas outside their locations whereas the wastewater sites are more localized.

Additionally, we identified two variants of the virus; Alpha (B.1.1.7) was identified in samples from the Ashanti and Greater Accra regions, while Delta (AY.36) was identified in the Eastern Region of Ghana. This is similar to studies from Israel and twenty European countries which reported the occurrence of B.1.1.7 [20, 21]. It is important to note that B.1.1.7 became a dominant SARS-CoV-2 strain in the UK a few months after its identification [22]. B.1.1.7 is a variant of concern and has been associated with lowered efficacy of vaccines and a rise in transmission [23, 24]. Several variants of SARS-CoV-2 including B.1.1, B.1.1.318, B.1.1.359, and B.1.1.7, B.1.617.2 have been identified in patient samples from communities across Ghana and in passengers entering the country [25, 26]. The presence of B.1.1.7 in humans and sewage in Ghana indicates the increased risk of infections, especially in vulnerable populations. Thus, control and management measures need to be enforced and adhered to reduce the risk of infections. A study in Brazil has reported the presence of Delta-related variant AY.36 in sampled patients [27]. Furthermore, in Nigeria, the Delta variant AY.36 caused the majority of COVID-19 cases although fewer cases worldwide are caused by AY.36 [28]. From the reports above, it seems the Delta variant AY.36 varies concerning its severity and spread. In Ghana, a study found the variant AY.36 to be 1% after nationwide surveillance [26]. However, further investigations will be necessary for Ghana to determine changes in its distribution in the country and the risk of infections.

In this study, we were unable to isolate viable SARS-CoV-2 from environmental sewage and wastewater by culturing in Vero cell lines. The failed attempt is similar to a study in South Korea that sought to isolate viable SARS-CoV-2 from the stool and urine of patients using Vero cell lines [29]. The difficulty in recovering viable viruses could be due to the low viral load concentrations of the samples which was evident based on the cycle threshold values (>35) obtained. It is also probable that the chemicals, pH changes, and high temperatures in the open drains and sewer lines could have inactivated and destabilized the virus [30], leaving behind only its genetic material which was detected. Another reason could be that the indigenous microbial population in the wastewater harmed the survival of the virus as reported by some studies [31, 32]. We however agree with the notion that detecting the viral genetic material in stool does not automatically mean that the viral particles are infectious [33, 34].

The general findings of this study are in line with reports that suggest that the virus occurs in sewage and wastewater worldwide [3, 4, 12, 35–37]. In Ghana, positive human cases were reported in all the sampled regions between 2020 and 2022 (https://ghs.gov.gh/covid19/dashboardm.php). Detecting the viral RNA in the sewage and wastewater collected during this period indicates that the virus was being shed in the waste matter of infected individuals. Hence, employing wastewater screening as an integral tool for SARS-CoV-2 environmental surveillance in the country could prove effective in establishing trends of COVID-19. Our success in detecting and sequencing variants of the virus from wastewater would be essential in formulating control efforts.

## 5. Conclusion

Our study showed that sewage and wastewater contained SARS-CoV-2 RNA. The viral RNA was identified in samples collected from all the sampled regions in Ghana. The two variants identified in this study were Alpha (B.1.1.7) and Delta (AY.36). These variants have been reported from clinical cases in the Ghanaian population indicating the sensitivity of sewage and wastewater surveillance. Although the calculated sample size could not be achieved due to inadequate resources and the positive samples that had high Ct values (>30) could not be sequenced successfully due to the specificity of the MinION sequencing platform, the findings from this study indicate the need to adopt control efforts to reduce the spread of the virus. It is recommended that there should be continuous education on the efficacy and safety of the COVID-19 vaccines to encourage the populations to accept and patronize them. Constant education and encouragement can go a long way to convince a majority of the population to get vaccinated and thus protect others from infections. Again, sewage and wastewater should be properly treated to prevent the potential spread of viable viruses which would negatively affect public health.

## Figures and Tables

**Table 1:** Distribution of SARS-CoV-2 and positivity rates across the environmental sites, 2020-2022.

Characteristics	Number of samples processed	Sites	SARS-CoV-2-positive samples	SARS-CoV-2 positivity rate (%)	*p* value
Total	354		59	17%	
Sample source					
Sewage	74		20	27%	0.013
Wastewater	280		39	14%	
Region					
Ashanti	57	Ahinsine	2	12%	0.03
		Asokore Mampong Zongo	5		
Bono	24	Sunyani Zongo	3	13%	
Bono East	25	Techiman Sisaline	2	8%	
Eastern	47	Akosombo	7	23%	
		Koforidua	4		
Greater Accra	102	Agbogbloshie	5	25%	
		Nima Freetown	7		
		Shiabu	9		
		University Of Ghana	5		
Northern	50	Koblimahgu	1	6%	
		Nyanshegu	2		
Volta	49	Aflao	1	14%	
		Hohoe	1		
		Kpando	1		
		Togbe Afede Road	4		

## Data Availability

All the data supporting this study are included in the article.
